# Taurine Induces Proliferation of Neural Stem Cells and Synapse Development in the Developing Mouse Brain

**DOI:** 10.1371/journal.pone.0042935

**Published:** 2012-08-20

**Authors:** Mattu Chetana Shivaraj, Guillaume Marcy, Guoliang Low, Jae Ryun Ryu, Xianfeng Zhao, Francisco J. Rosales, Eyleen L. K. Goh

**Affiliations:** 1 Program in Neuroscience and Behavioral Disorder, Duke-NUS Graduate Medical School, Singapore, Singapore; 2 Cognition Center of Excellence, Abbott Nutrition Research & Development, Asia Pacific Center, Singapore, Singapore; 3 Department of Physiology, Yong Loo Lin School of Medicine, National University of Singapore, Singapore, Singapore; 4 A*STAR-Duke-NUS Neuroscience Research Partnership, Proteos, Singapore, Singapore; University of Texas Health Science Center at San Antonio, United States of America

## Abstract

Taurine is a sulfur-containing amino acid present in high concentrations in mammalian tissues. It has been implicated in several processes involving brain development and neurotransmission. However, the role of taurine in hippocampal neurogenesis during brain development is still unknown. Here we show that taurine regulates neural progenitor cell (NPC) proliferation in the dentate gyrus of the developing brain as well as in cultured early postnatal (P5) hippocampal progenitor cells and hippocampal slices derived from P5 mice brains. Taurine increased cell proliferation without having a significant effect on neural differentiation both in cultured P5 NPCs as well as cultured hippocampal slices and in vivo. Expression level analysis of synaptic proteins revealed that taurine increases the expression of Synapsin 1 and PSD 95. We also found that taurine stimulates the phosphorylation of ERK1/2 indicating a possible role of the ERK pathway in mediating the changes that we observed, especially in proliferation. Taken together, our results demonstrate a role for taurine in neural stem/progenitor cell proliferation in developing brain and suggest the involvement of the ERK1/2 pathways in mediating these actions. Our study also shows that taurine influences the levels of proteins associated with synapse development. This is the first evidence showing the effect of taurine on early postnatal neuronal development using a combination of *in vitro*, *ex-vivo* and *in vivo* systems.

## Introduction

The development of the nervous system involves the coordination of specific cellular events including proliferation, differentiation, migration, outgrowth of axons and dendrites, formation of synapses, myelination, and programmed cell death. Chemical-mediated disruption of one or more of these processes can potentially disrupt the function of the nervous system [1], [2]. Synaptic plasticity has also been known to be closely associated with neural system development [3] and has been defined as the modification of synapses structurally and functionally by different stimuli and environmental cues such as activity and intrinsic determinant [4]. The hippocampus plays important roles in long-term and spatial memory, storage, retrieval and navigation [5]. Newly generated neural precursor cells from the sub-granular proliferative zone at the DG migrate to regions of differentiation, where they grow, develop and become matured neurons [6], [7]. In becoming matured, functionally integrated neurons, the newborn cells must first acquire the ability to form dendrites and synapses, to receive synaptic contacts and to extend axons, processes [6], [8] that are influenced by many factors. Compound absorbed through the diet have long been touted to affect postnatal neural development and neurogenesis [9]. A study showed that a food product derivative, apigenin stimulates neurogenesis in the mice hippocampus by promoting neuronal differentiation, and also enhanced learning and memory [10]. Neuroactive compounds such as cocaine and cannabinoids were also shown to alter the proliferation and differentiation rates of NPCs and result in subsequent neurodevelopment and neurocognitive deficits [11], [12].

Taurine (2-aminoethanesulfonic acid) plays an important role in several essential biological processes such as development of the central nervous system and the retina, reproduction, immune-modulation, osmoregulation, and membrane stabilization [13], [14], [15]. Endogenous taurine is derived from cysteine. The brain synthesizes only a limited amount of taurine, and most taurine synthesis occurs in the liver [16]. Taurine is suggested to have an important role in brain development, as its level is 3–4 times higher in developing and neonatal brain than adult brain [17]. This age related decline is a consistent feature observed among species, regardless of their original differences in taurine concentration [18]. Studies in monkeys fed with dietary formulas without taurine showed prominently a defective organization of cortical layers in the visual cortex [15]. Cats born from taurine deficient mothers have smaller brain weight and an abnormal morphology in the cerebellum and the visual cortex. Delayed migration of neuroblasts and glioblasts is also observed in the visual cortex. Pyramidal cell number is reduced and neurons show poor arborization in taurine deficient kitten [19], [20]. These studies further demonstrate the importance of taurine in the developing brain. Taurine is also reported to increase or restore cell proliferation of human fetal neurons [21] and has been postulated to influence neurotransmission [22]. Altogether, these findings point to taurine being essential for optimal proliferation, development and maturation of brain cells. The main objective of the present work is to determine the potential effect of taurine on neural stem/progenitor cell proliferation and neurogenesis in the developing brain, using cultured neural progenitor cells, and cultured hippocampal slices representing *in vitro*/*ex-vivo* models of early post natal neurogenesis and also in embryonic hippocampus of taurine supplemented embryos. In addition, we investigated the mechanism of the taurine effect on neural stem/progenitor cell proliferation. We also studied the effect of taurine on neurite growth and synaptogenesis in cultured embryonic hippocampal primary neurons.

## Results

### Taurine Enhances Proliferation of Neural Stem/progenitor Cells (NPC)

**Figure 1:**
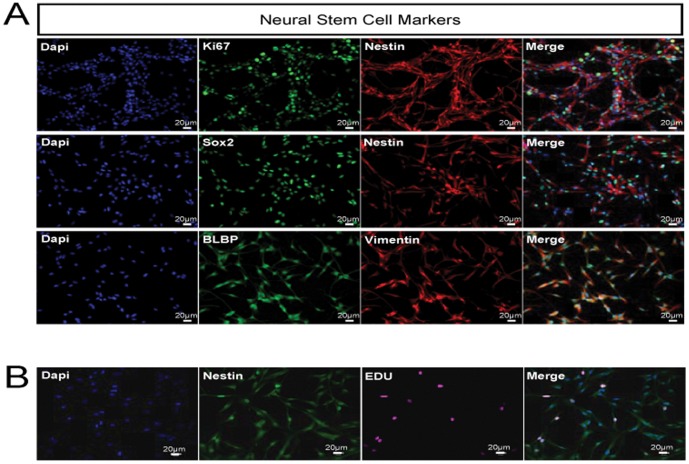
Neural progenitor cells (NPCs) isolated from 5 days old mouse hippocampus are multipotent. These NPCs can proliferate and self-renew in culture, as demonstrated by the expression of proliferation marker, Ki67 and the standard NPCs markers, Nestin, Sox2, BLBP and Vimentin (A). All DAPI positive cells are positive for Nestin and the dividing cells are labeled with EdU (B). Scale bar = 20 µm.

The capacity for self-renewal and continuous proliferation is one of the key characteristics of stem/progenitor cells *in vitro* [23]. To evaluate the effect of taurine on NPCs, we isolated NPCs from hippocampus of 5 days old mice. NPCs were characterized based on their ability of self-renewal, expression of NPC markers as well as their ability to differentiate to neuronal and glial populations. All the cells express the standard NPC markers such as Nestin, Sox2, BLBP and Vimentin. They also express the proliferation marker Ki67 and their expression is constant regardless of the passage number (Fig. 1A).

**Figure 2:**
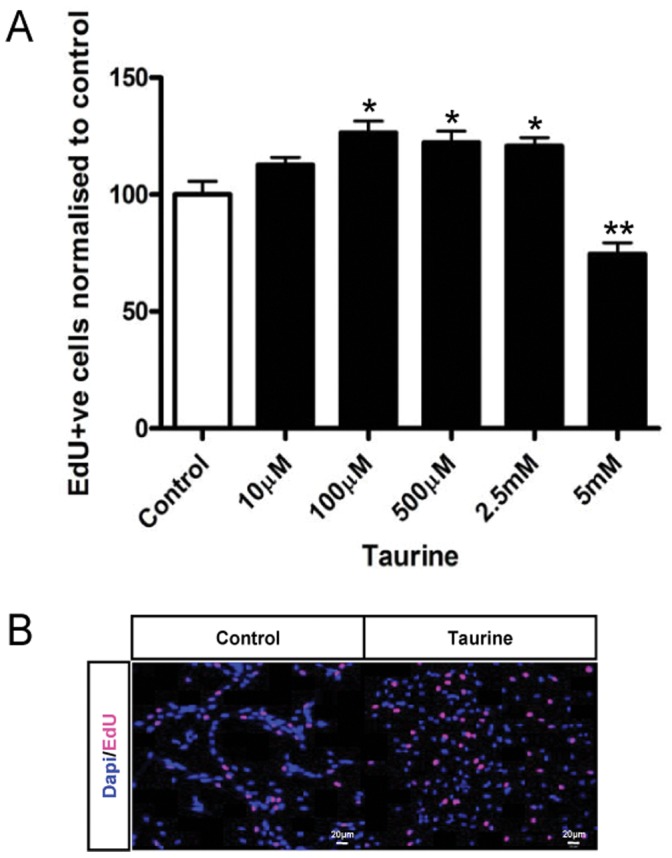
Effects of taurine treatment on proliferation of P5 hippocampal stem/progenitor cells. Cells were treated with five different concentrations (10 µM, 100 µM, 500 µM, 2.5 mM and 5 mM) of taurine for 48 h and labeled with EdU (10 µM) in the last 3 h of incubation. Data are presented as percentage of EdU positive cells normalized to control (A). Data are expressed as mean ± SEM. * *P*<0.05 and ** *P*<0.01. Representative microscopic images showing EdU-labeled P5 hippocampal progenitor cells in control (*left panel*) and taurine treated groups (*right panel*)) (B). Scale bar = 20 µm.

In order to determine the effects of taurine on NPC proliferation, we treated the cells with different concentrations of taurine (10 µM, 100 µM, 500 µM, 2.5 mM and 5 mM) and labeled the dividing cells with 5-ethyl-2′-deoxyuridine (EdU). All cells on the coverslips are positive for DAPI and Nestin, and the dividing cells are labeled with EdU (Fig. 1B). The number of EdU positive cells in 100 µM taurine -treated group increased significantly by 28% (*P*<0.05) as compared to the control. A 20% increase was also observed when treated with 500 µM or 2.5 mM taurine. (Fig. 2A–B, *P*<0.05), indicating that taurine at appropriate concentrations could stimulate the proliferation of P5 NPCs. However, taurine at a lower concentration of 10 µM did not induce any significant effect on NPC proliferation and at higher concentration (5 mM), the number of NPCs decreased (*P*<0.01) as compared to control.

**Figure 3:**
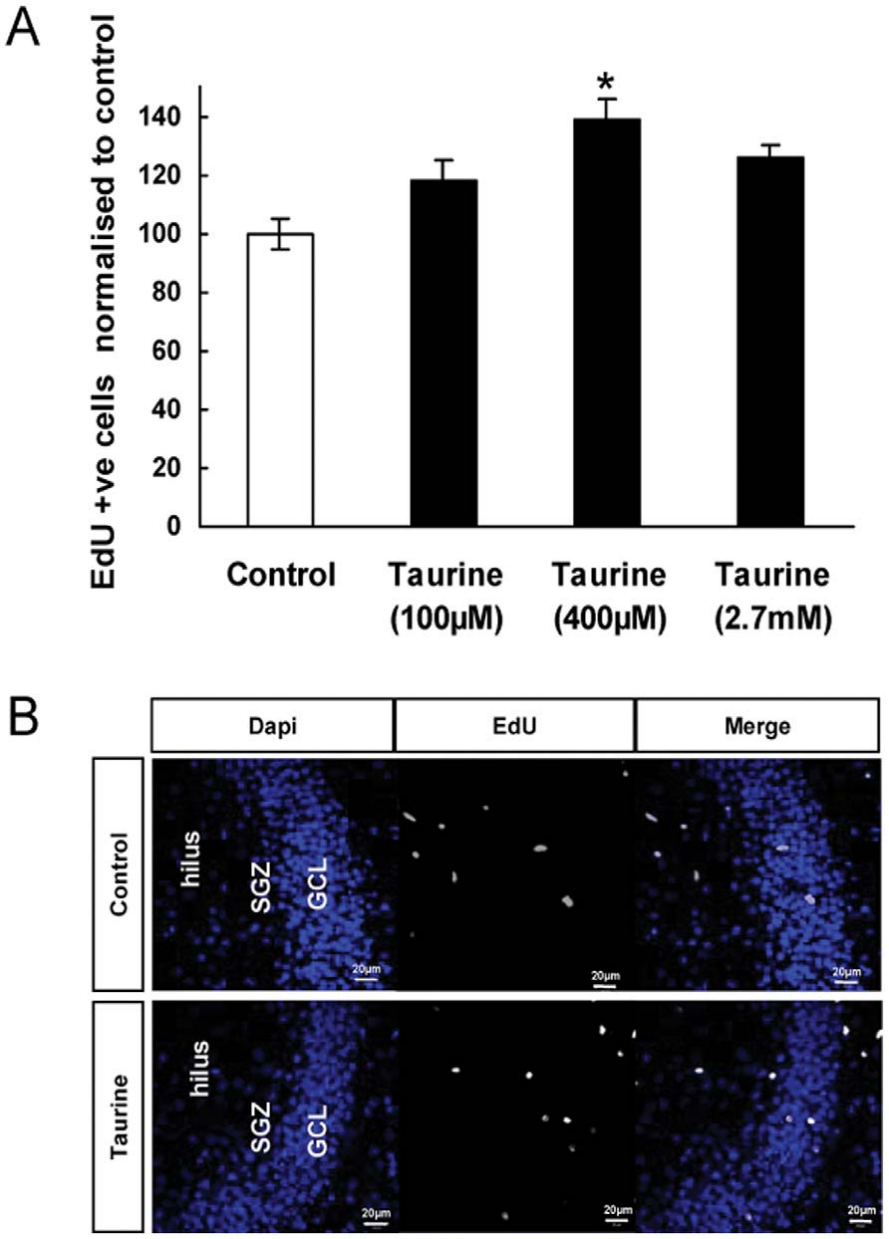
Effects of taurine treatment on cell proliferation in the dentate gyrus of cultured hippocampal slices. EdU labeling was used to assess cell proliferation. The culture medium was changed to serum free condition on day 7 and taurine was added to the medium on day 9. EdU (10 µM) was added to the culture medium at day 11 and the slices were fixed and processed for EdU staining on day 17. Numbers of EdU positive cells in dentate gyrus were analyzed and the data are presented as mean ± SEM. (* *P*<0.05) (A). Images showing EdU-labeled cells in the dentate gyrus of control (upper *panel*) and taurine treated groups (lower *panel*) (B). SGZ and GCL denote subgranular zone and granule cell layer respectively. Scale bar = 20 µm.

**Figure 4:**
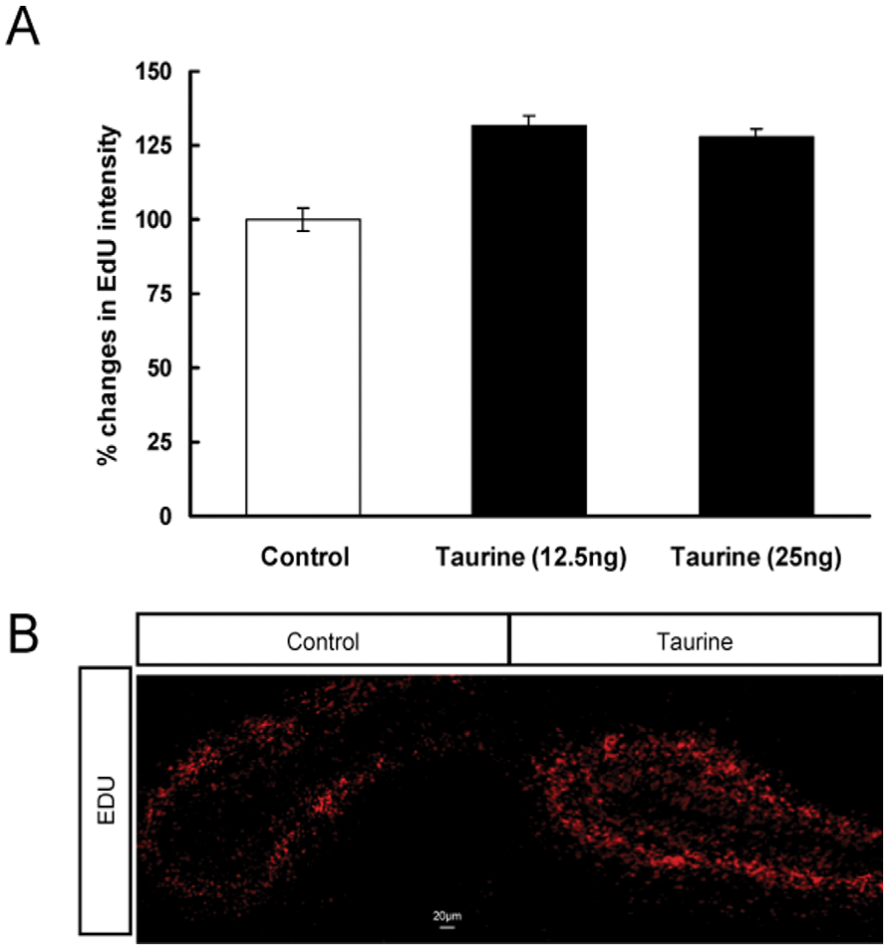
Effects of taurine treatment on cell proliferation in the dentate gyrus of embryonic hippocampus. The embryonic brains were fixed at E-17. EdU intensity was measured for 6–7 identical sections per brain and at least 5–6 fetuses were used. The data of the percentage changes in EdU intensity are presented as mean ± SEM. (A). Representative images showing EdU-labeled cells (red) in the dentate gyrus of control (*left panel*) and taurine treated groups (*right panel*) (B). *P*>0.05, Scale bar = 20 µm.

EdU labeling was also used to determine the proliferation of cells in the dentate gyrus of cultured hippocampal slices. The total number of EdU positive cells in taurine (400 µM) treated group was significantly more (by 38%) as compared to the control group (Fig. 3A–B, *P*<0.05), indicating that taurine stimulates the proliferation of neural stem cells in the dentate gyrus of the developing brain. Targeting of nutritional compounds to the embryonic brains during the prenatal developmental periods could lead to a more complete understanding of the effect of these compounds on the developing brain in vivo. Therefore, we evaluated the effects of taurine on stem/neural progenitor cell proliferation in the embryonic hippocampus *in vivo* via in utero microinjection. In these experiments, taurine was injected directly into ventricles of the embryonic stage 13 (E13) fetal mice brains. The embryonic stage E13 was selected because it is just before the expected peak of cell division and migration in the hippocampus. Dividing cells were labeled with EdU, and these EdU positive cells in the developing hippocampus were analyzed by quantifying the fluorescence intensity of EdU in hippocampal slices from pups that were sacrificed and fixed at E17. We found that taurine stimulated a 31% increase (*P*>0.05) in the EdU intensity as compared to control (Fig. 4A, B).

### The Effect of Taurine on the Differentiation of Cultured P5 Stem Cells/Neural Progenitor Cells to Specific Lineages

**Figure 5:**
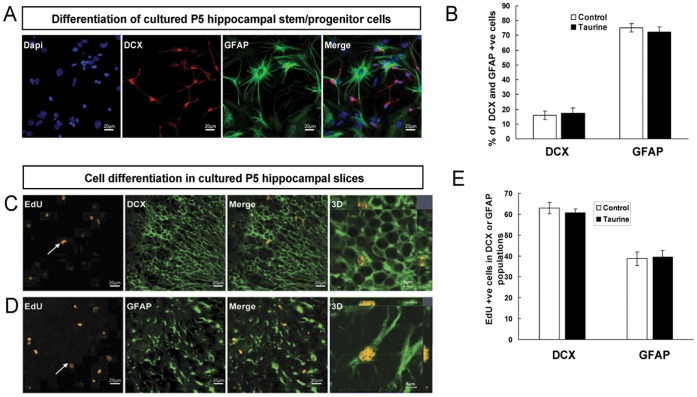
Effects of taurine treatment on cell differentiation. EdU labeling and differentiated cell markers (DCX labels neurons and GFAP labels astrocytes) were used to assess the differentiation and fate choice of newly generated cells in NPCs and in DG of cultured hippocampal slices. Representative images of cultured P5 hippocampal stem/progenitor cells treated with taurine (100 µM) and allowed to differentiate for 5 days before fixation for immunohistochemical processing (A). Quantitative analysis of cells positive for EdU and DCX or GFAP indicating that the percentage of DCX or GFAP was not altered by taurine treatment (B). Representative images showing cells positive for EdU and DCX or GFAP in DG of hippocampal slices (C and D). Quantitative analysis of cells positive for EdU and DCX or GFAP in the dentate gyrus indicating that the percentage of DCX or GFAP was not altered by taurine treatment (E). All quantitative data are expressed as mean ± SEM. Scale bar = 20 µm.

Next, we determined if taurine affects the differentiation of hippocampal stem/progenitor cells *in vitro*. NPCs were induced to differentiate in the differentiation media for 5 days that contains a lower concentration of FGF-2 and no EGF as published previously [24]. The low concentration of FGF-2 is essential for survival of our P5 NPCs and does not impair the differentiation efficacy. After 5 days of differentiation, the number of Ki67 and Nestin positive cells decrease dramatically from 100% to less than 8% (data not shown) and these P5 NPCs can be differentiated into doublecortin- (DCX) expressing neurons and glial fibrillary acid protein- (GFAP) expressing astrocytes (Figure 5A). Taurine treatment showed no significant effect on the percentages of cells that were positive for the neuronal marker, DCX or the glial marker, GFAP (Fig. 5B).

To determine if taurine treatment can affect the fate of newborn cells in organotypic brain slices, EdU-labeled cells were analyzed 6 days after EdU was added to hippocampal slices treated with taurine or vehicle. This length of time is known to be sufficient for newly proliferated EdU-labeled cells to differentiate into their different lineages. The differentiation of EdU-labeled cells was identified through immunohistochemistry using specific antibodies against the neuronal marker, DCX, or the glial marker, GFAP (Fig. 5C–E). Analysis of co-localization of EdU with DCX or GFAP indicated that neither the percentages of DCX nor GFAP expressing cells were significantly altered by taurine treatment. These *ex vivo* findings are consistent with our results from the earlier *in vitro* studies showing no significant effects of taurine on the differentiation of P5 NPCs (Fig. 5B).

### Taurine-induced Proliferation is Dependent on the Phosphorylation of p44/42 MAPK

**Figure 6:**
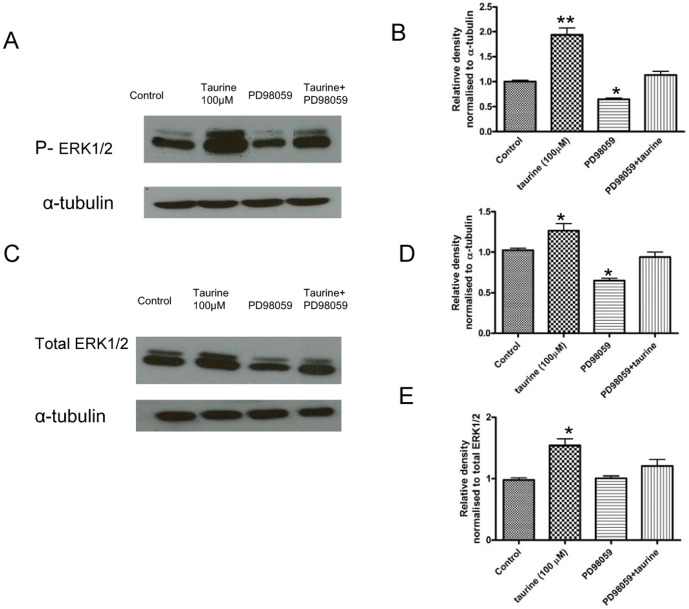
ERK1/2 is involved in taurine induced NPC proliferation. The cells were incubated with or without 100 µM taurine for 48 hrs. ERK1/2 inhibitor, PD98059 was added to the media 30 min before taurine treatment. Representative immunoblots showing protein levels of phosphorylated ERK1/2 (A) and total ERK1/2 (C). Graphs showing the ratio of densitometric measurements of phosphorylated ERK1/2 and total ERK1/2, normalized with total protein levels of α-tubulin. (B and D). Graph showing fold change in phosphorylated ERK1/2 upon treatments as indicated, normalized with total ERK1/2 (E) * *P*<0.0 5 and ** *P*<0.01 as compared with the control. The western blots are representative blots from at least three individual experiments.

The p44/42 MAPK (ERK1/2) growth signaling pathway has been shown to be involved in many different cellular processes and is essential for proliferation of NPCs [25]. To determine if taurine activates this ERK1/2 signaling pathways in P5 hippocampal stem/progenitor cells, phosphorylation of ERK1/2 was evaluated by western blot analysis following taurine treatment. Taurine treatment dramatically elevated the level of phosphorylated ERK1/2 (*P*<0.01, Fig. 6A and B) and a modest increase in the total ERK1/2 (*P*<0.05, Fig. 6C and D). Pretreatment with ERK1/2 inhibitor, PD98059 inhibited the taurine-stimulated increase in phosphorylation of ERK1/2. We normalized the levels of phosphorylated ERK1/2 by levels of total ERK1/2 proteins (Fig 6 E) and our results showed that pretreatment with PD98059 specifically inhibited taurine-stimulated increase in phosphorylation of ERK1/2. These results suggest that ERK1/2 signaling pathways are activated by taurine in hippocampal progenitor cells.

**Figure 7:**
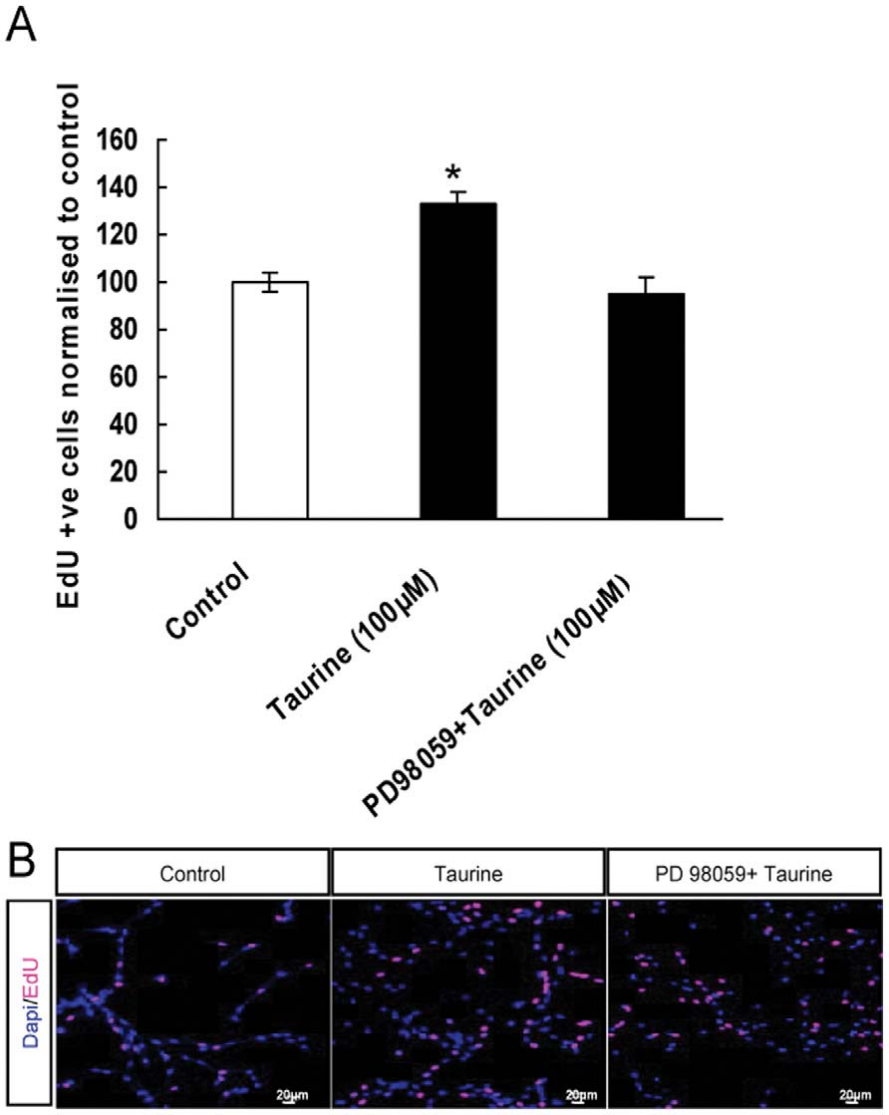
Taurine-induced proliferation of P5 hippocampal stem/progenitor cells is dependent on the activation of ERK1/2. Cultured hippocampal stem/progenitor cells were incubated with ERK1/2 inhibitor PD98059 for 30 min followed by treatment with taurine for 48 h and labeling with EdU (10 µM) in the last 3 h of incubation. Quantitative data presented as percentage of EdU positive cells normalized to control, indicates pretreatment with ERK1/2 inhibitor attenuates the taurine induced increase in the number of EdU-labeled cells (A). Representative images showing EdU -labeled P5 hippocampal progenitor cells in control (*left panel*), taurine treated groups (centre), and ERK1/2 inhibitor + taurine (*right panel*) (B). Scale bar = 20 µm. All data presented here are expressed as mean ± SEM. * *P*<0.05.

To further determine whether activation of the ERK1/2 signaling pathway mediates the action of taurine on cell proliferation, P5 hippocampal stem/progenitor cells were exposed to the ERK1/2 inhibitor PD98059 (10 µM) followed by taurine treatment. The pretreatment with the ERK1/2 inhibitor PD98059 diminished the taurine-induced effect on proliferation (Fig. 7A–B). These results further suggest that activation of ERK1/2 is involved in the stimulatory effect of taurine on NPC proliferation.

### The Effect of Taurine on Neuritogenesis by NPC Differentiated Neurons and Rat Embryonic Primary Neurons

**Figure 8:**
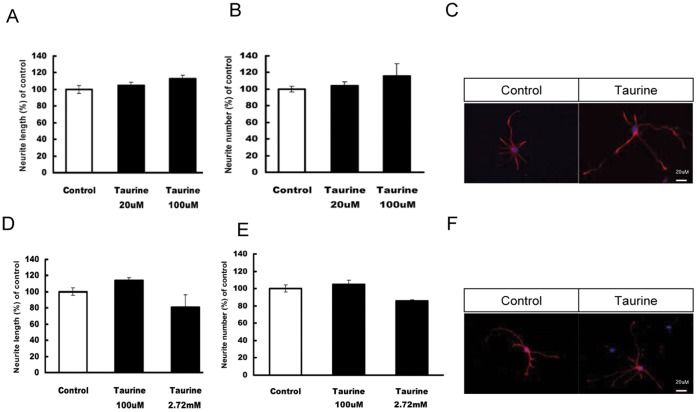
The effects of taurine on neurite outgrowth of NPCs and primary neurons. The average total neurite length (A and D) and neurite numbers (B and E) were measured. Images in C and F show representative primary neurons and NPCs treated with vehicle (*left panel*) or taurine (*right panel*) respectively. Taurine was added to the media on day 2. Primary neurons and NPCs were then fixed on day 5 and 7 respectively. Differentiated neurons were then analyzed and data presented as mean ± SEM. Taurine shows a small non-significant effect on neurite development of primary neurons as well as newborn neurons. Scale bar = 20 µm.

To further investigate the effects of taurine on neuronal development, we studied the effect of taurine on neurite growth in neurons differentiated from P5 neural progenitor/stem cells and cultured E18 rat embryonic primary neurons. We found that taurine has a moderate influence on neurite growth. It modestly increased the neurite length in NPCs (16%) and in primary neurons (18%). However, no significant difference in neurite number was observed between control and treated cells (Fig. 8A–F). Higher concentration of taurine (for example, at 2.7 mM) significantly decreased the neurite length and number of neurite in neurons differentiated from NPCs, therefore, we used only lower concentration (100 µM) to check on its effect on primary neurons.

### Taurine Increased Synaptic Proteins Expression in Hippocampal Primary Neurons and Cultured Hippocampal Slices

**Figure 9:**
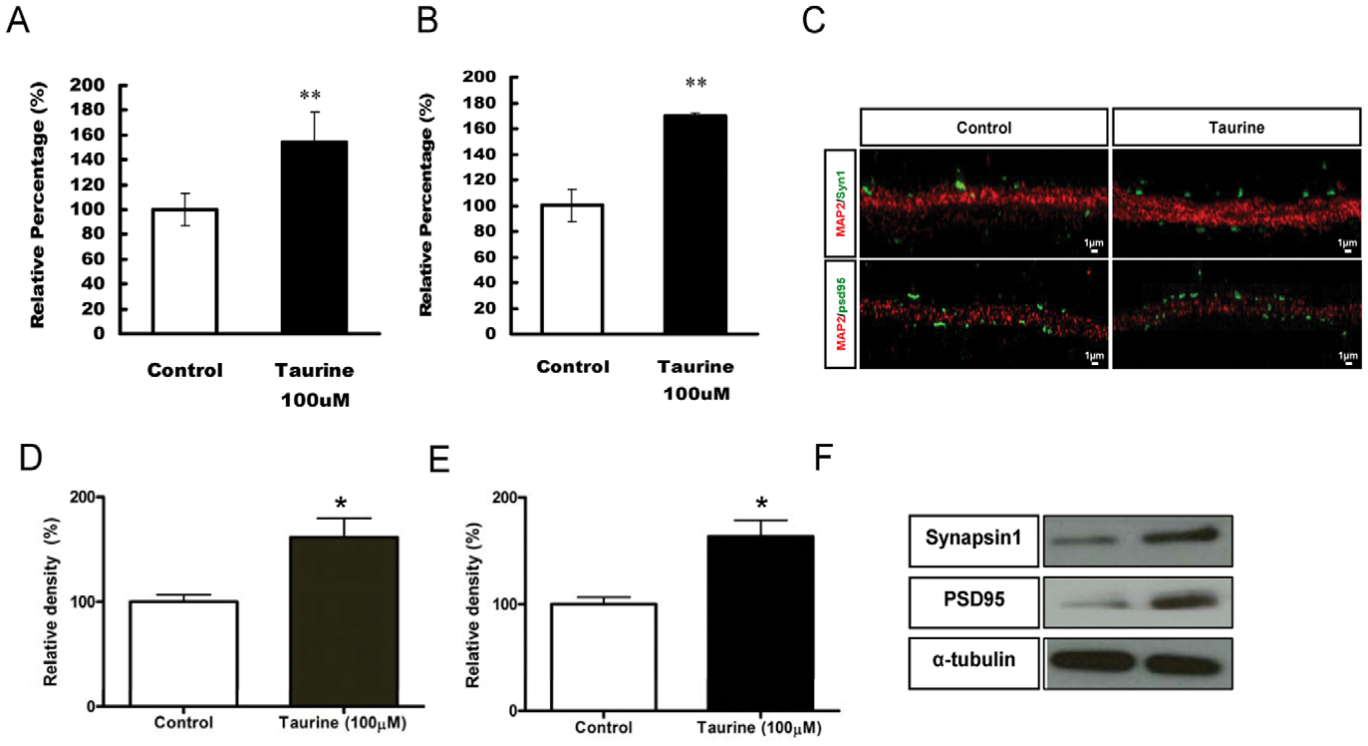
The relative percentage of the effect of taurine on synaptic puncta and protein levels. Taurine was added to the primary neurons on day 2 and the neurons were then fixed and processed at day 9 and 15. The quantification of synapsin 1 and PSD 95 positive puncta was done by measuring the number of puncta per µm. Data are presented as percentages normalized to the control (100%) ± SEM and puncta from at least 20 different neurons of each batch from at least three repeated experiments were quantified. Taurine treatment significantly increased the number of Synapsin 1 (A) and PSD 95 (B) puncta. Representative images show synapsin 1 (*top panel*) and PSD 95 puncta (*bottom panel*) respectively (C). The protein levels of synapsin 1 (D) and PSD95 (E) in protein lysates in primary neurons were shown using western blotting analysis (D-F). Data were normalized with total protein levels of α-tubulin. * *P*<0.05 and ** *P*<0.01. Scale bar = 1 µm.

To determine if taurine induces synaptogenesis in the neurons of the brain, we added taurine (100 µM) to cultured hippocampal primary neurons from 2 to 15 days *in vitro* (DIV). At the end of the incubation period, the neurons were immunostained using an antibody against PSD-95, an excitatory postsynaptic marker. Taurine treatment led to 60% increase (*P*<0.01) in the density of PSD-95 positive puncta (Fig. 9B, C). We also immunostained for synapsin 1, the marker of the presynaptic terminal, at the end of 9 DIV and also observed a significant increase (*P*<0.01) in the density of synapsin 1 positive puncta after taurine treatment (Fig. 9A, C). We also determined the protein expression levels of synapsin 1 and PSD 95 using western blot analysis and observed an increase in expression of synapsin 1 (Fig 9D, F, *P*<0.05) and PSD 95 (Fig. 9E, F, *P*<0.05). The increased expression levels of PSD-95 and synapsin 1 indicate that taurine also has important role in synaptogenesis.

## Discussion

Our results documented the importance of taurine in the developing brain cells by enhancing proliferation and synaptogenesis. We demonstrated in this study that taurine supplementation induces proliferation of cultured P5 neural stem cell/progenitor cells, and stem cells in dentate gyrus of cultured organotypic hippocampus slices derived from P5 mice, signifying the effect of taurine in early postnatal development. It has been reported that monkeys fed with formulas without taurine showed a prominent defect in organization of cortical layers in the visual cortex [26]. Cats born from taurine-deficient mothers have smaller brain weight and an abnormal morphology in the cerebellum and the visual cortex [19], [20]. Delayed migration of neuroblasts and glioblasts is also observed in the visual cortex [15]. Pyramidal cell number is reduced and cells show poor arborization in taurine deficient kitten [19], [20]. These studies further demonstrate the importance of taurine in the developing brain. During the perinatal period, progenitors of neurons and glial divide and migrate to reach their final destinations within various brain regions, creating the structures of the brain and setting the stage for brain function or dysfunction later in life. During this period, brain development is sensitive to the supply of essential nutrients. Therefore, our finding that taurine influences stem cell proliferation, could potentially explain that smaller brain weight and abnormal brain morphology of taurine deficient mouse could be due to decreased proliferation of neural progenitor cell in the absence of taurine.

Direct delivery of nutritional compounds targeting into embryonic brain regions with nutritional compounds during prenatal developmental periods allow a more complete understanding of the effect of these compounds on developing brain. *In utero* microinjection techniques enable the delivery of compounds directly to the embryo brain and examine neurodevelopment during and after embryonic developmental stages. Targeting of specific brain regions with tracers during developmental periods can be combined with immunohistochemical methods and the administration of pharmacological treatments. Although this approach has the advantage of providing a better understanding on how taurine may work *in vivo* at a physiologically relevant environment, this could also be a disadvantage for the interpretation of results. The microenvironment and neighboring cells may or may not condition the effects of taurine. Moreover, the delivery of the appropriate concentration of taurine to the target destinations and cells is also a challenge. Nonetheless, this type of study could still potentially lead to a more complete understanding of the cellular and molecular mechanisms that regulate perinatal pharmacology [27]. Therefore, we injected embryonic mouse with taurine to the embryos on day E13, just before the expected peak of proliferation and migration in hippocampus. Taurine injected mouse showed an increased in EdU labeling, suggesting increased proliferation of stem cells. However, the difference between vehicle and taurine treated groups is less than those observed in the *in vitro* and *ex-vivo* experiments where taurine is delivered directly to the cells. This could be due to that embryos may already have enough taurine through the mother and further supplement may not cause dramatic changes. It could also be that insufficient amount of taurine from ventricles (site of delivery) may have reached hippocampus. Our study also supports earlier reports that taurine increases or restores cell proliferation of human fetal neurons [21] and embryonic cultured NPCs [28].

While most of the earlier studies emphasized the importance of taurine during embryonic development, the novelty of our studies is that taurine increases the neural progenitor cell proliferation in cultured P5 hippocampal slices, as well as NPCs derived from P5 mice. This highlights the importance of taurine during early postnatal brain development. A significant increase in the number of newborn cells was observed in taurine treated slices indicating increased neural progenitor cell proliferation suggesting that a relatively short exposure, at early life stages, has a potentially lasting impact on neuron production. In the dentate gyrus of mice and rats, neurogenesis mainly takes place in the first two postnatal weeks and then gradually decreases [29]. Using hippocampal slices, we could demonstrate that taurine treatment within a time window of intense granule cell proliferation, during early postnatal development, notably enhanced granule cell formation. The dentate gyrus belongs to the major network of the hippocampal region, the entorhinal–dentate–CA3–CA1–entorhinal system. Signal processing by this network is essential for establishment/retrieval of long-term memories. Since newborn granule cells are preferentially incorporated into spatial memory networks [30], [31], it is expected that the prominent increase in granule cell number induced by early exposure to taurine is accompanied by enhancement of memory functions played by the hippocampal region. Moreover, clinical evidence suggests a specific effect of taurine on cognitive development. Warthon *et al.* [32], found that low plasma taurine concentrations in preterm and small-for-gestational age neonates are associated with lower scores on the Bayley mental development index at 18 months and the Wechsler Intelligence Scale for Children-revised arithmetic subtest at 7 years. The authors did not specify a mechanism for this selective effect of taurine. However, they noted that taurine’s concentrations are differentially distributed across brain tissues, supporting taurine’s selective effect. In this case, the hippocampus is likely, as others have indicated that it is a “metabolically needy” organ because of its high sensitivity to nutrient and energy alterations [33].

MAPK/ERK can be activated by many signals including mitogenic signals, cytokines, and growth factors. The MAPK/ERK-dependent pathways play a key role in regulating cell proliferation and differentiation [25]. Previous studies have showed that MAPK is involved in the proliferative effects of taurine in osteocytes [34]. We demonstrated that taurine supplementation increased ERK1/2 phosphorylation, indicating a role of this pathway in taurine induced NPC proliferation. The ERK inhibitor (PD98059) causes significant inhibition of proliferation in taurine treated NPCs further supporting that taurine-mediated ERK1/2 phosphorylation increases proliferation of NPCs. Taurine’s proliferative effect on NPC occurs in addition to that of the growth factor effects from the growth media. To the best of our knowledge, this is the first report demonstrating taurine induces proliferation in neural progenitor cells through activating ERK1/2 pathway.

Cultured NPCs differentiate under appropriate conditions into neurons and glia [35]. However, taurine did not significantly increase the number of neurons or astrocytes differentiated from NPCS, though a modest 8% increase in the number of neurons was observed in cultures containing taurine. Recently, Hernández-Benítez *et al.* [28] also reported that taurine did not change the number of embryonic NPCs differentiated to neurons, but the authors found an increase in NPCs differentiated into astrocytes (GFAP-positive cells). These effects may depend on the age of embryos that the progenitor cells were derived from, as they have used embryonic NPCs instead of the P5 NPCs that we are using in our study, and possibly also dependent on the cell culture conditions [36], [37].

Some studies have shown that taurine influences neurotransmission [22]. Our results are in agreement with this notion as taurine can influence the neuronal maturation and synaptogenesis. Taurine has moderate effects on the number and length of neurite branches in primary neurons as well as NPCs-derived neurons. Taurine supplementation increases the expression of presynaptic and postsynaptic proteins in cultured hippocampal primary neurons as well as in hippocampal slices. Synapsin 1, a family of neuron-specific phosphor-proteins associated with the membranes of synaptic vesicles, have been identified as a molecular component involved in synaptogenesis [38]. Taurine treatment in hippocampal neurons significantly increased the number of synapsin 1 puncta (synapsins associated with synaptic vesicles) and synapsin 1 protein expression level, which is indicative of improved synaptogenesis. The postsynaptic density–95 (PSD-95) is a dendritic membrane-associated protein and may participate in synapse development, because it clusters at synapses before other postsynaptic proteins [39]. Husseini *et al*. [40], found that over expression of PSD-95 in hippocampal neurons can drive maturation of glutamatergic synapses. They also reported that postsynaptic expression of PSD-95 enhances maturation of the presynaptic terminal. Increased PSD-95 expression also increases the number and size of dendritic spines. These results demonstrate that increased PSD-95 levels in taurine treated neurons can orchestrate synaptic development and are suggestive of roles for taurine in synapse stabilization and plasticity.

Our data support the notion that taurine is necessary for normal growth, development and maturation of neurons. It is well established that the levels of learning and memory are associated with the levels of neurogenesis and the number and complexity of dendrites. Taurine also shows moderate effect on dendrite growth and increase synaptogenesis. These studies explain the importance of taurine on maturation of the neurons. Together, these findings suggest possible effects of taurine in different stages of brain development, playing the role of a factor necessary for optimal maturation of brain cells. Taurine is present at high levels in the retina of many vertebrates [41]. Taurine is known to be involved in the mediation of multiple functions, such as osmoregulation, modulation of calcium fluxes, neuromodulation, protection from oxidative stress, modification of protein phosphorylation, membrane stabilization, affectation of cell migration in the brain and in the retina, regulation of axonal outgrowth, elevation in the number of regenerating retinal cells after nerve lesion [42]. Here, we showed an important role of taurine in increasing neural progenitor cell proliferation and neuronal maturation in the developing brain, which explains the importance of high taurine levels in the developing brain compared to adult brain [18], [43]. These results indicate that taurine is an important nutritional factor for neurogenesis and neuronal development. Whittle *et al.* [44] recently reported that fetal Down syndrome brains showed reductions in the levels of serotonin, taurine, and dopamine in the frontal cortex. Juveniles passerine birds fed taurine-rich diets as neonates matures into much larger risk takers and more adept at spatial learning tasks This also explains the importance of taurine in brain development. Taurine’s effects on the neurons suggest their functions in enhancing synaptic plasticity and enhancing learning and memory. It is well known that hippocampal neurogenesis and neuritogenesis is accompanied with a better spatial learning performance. Thus, this study provides evidence that increased proliferation and neuritogenesis in the hippocampus may be a part of a basis for the beneficial effect of taurine on behavioral performance.

## Materials and Methods

### Neural Progenitor Cultures

NPC isolation was performed according to established protocols [45], [46]. Briefly, 5 days old C57/BL6 mice were sacrificed and then the hippocampus, as a whole, was gently separated from the corpus collosum. The tissue samples were diced with a scalpel blade in Hanks’ Balanced Salt Solution (HBSS) (Invitrogen) and centrifuged at 2000 rpm for 5 min. After removing the supernatant, tissue samples were enzymatically digested with Papain (Worthington) containing DNase I 250 U/ml and Dispase II 1 U/ml at 37°C for 30 min. Tissues were then dissociated into single cells and centrifuged to obtain cell pellet. The pellet was resuspended into Dulbecco’s Modified Eagle Medium (DMEM)/nutrient mixture F-12 (Invitrogen), supplemented by 1% N2 supplement (Invitrogen), 1% penicillin/streptomycin and 1 mM l-glutamine. Finally, cells were washed and resuspended in culture media supplemented with FGF-2 (R&D Systems) and EGF (Merck), both at 20 ng/ml in 5 mg/ml heparin. Primary hippocampal cells were incubated for 7 days to allow neurosphere formation. The neurospheres were then dissociated using accutase and NPCs were cultured as monolayer on laminin (10 µg/ml) coated plates. The NPCs are characterized by staining for proliferation and stem cell markers (Figure 1). Cells are harvested and seeded at a density of 20,000/cm^2^ per well on 12 mm coverslips precoated with laminin. The cells were treated with taurine for 48 hrs. To measure whether taurine supplementation promotes NPC proliferation, 10 uM of thymidine analogue, 5 ethynyl-2′-deoxyuridine (EdU) (Invitrogen) was added after 45 hrs for 3 hrs and fixed with paraformaldehyde (PFA). The cells were stained for EdU using EdU staining kit according to manufacturer’s instructions (Invitrogen). EdU positive cells were then counted using cell profiler software. Figure 1B shows that all DAPI positive cells are nestin positive and the proliferating cells are labeled with EdU. Data were analyzed by one-way ANOVA with Dunnet’s post hoc test. *P*<0.05 is considered as significant.

To determine whether ERK1/2 signaling pathway mediates the action of taurine on cell proliferation, P5 hippocampal stem/progenitor cells were exposed to the ERK1/2 inhibitor PD98059 (10 µM) followed by taurine treatment. The data were analysed by one-way ANOVA followed by Dunnett’s Post-hoc test.

For differentiation experiments, cells were seeded onto poly-L-ornithin and laminin coated coverslips at a density of 25,000/cm^2^ per well in 24 well plates in defined medium composed of DMEM/F-12, N2 supplement and B27 supplement (Invitrogen), 2 mM glutamine and 2 µg/ml pen/streptomycin, supplemented with fibroblast growth factor (5 ng/ml). Taurine was added to the media and the medium was changed every alternate day for 5 days. The cells were then fixed with PFA for 30 min, washed with PBS and processed for immunocytochemistry. Data were analyzed by t-test. *P*<0.05 is considered as significant. For biochemical analysis, NPCs were cultured in 6 cm plates, treated with taurine for 2 days, and then processed for western blot analysis.

### Primary Neuronal Cultures

Ethics statement: All procedures involving mice and rats were in accordance with IACUC guidelines.

Hippocampal neurons were isolated from the hippocampus of embryonic day 18 Sprague-dawley rat embryos as previously described [47]. Briefly, hippocampus were dissected from E18 rat brains under sterile conditions in EBSS (Gibco) containing 10 mM HEPES and the hippocampal tissue was dissociated in papain and passed through a fire polished Pasteur pipette and resuspended in MEM (GIBCO supplemented with 0.2 µM fetal calf serum, 100 U/ml penicillin, 100 µg/ml streptomycin and N2 supplement (GIBCO). Dissociated neurons were cultured on poly-l-lysine coated plates or coverslips at a density of 2.5 × 10^4^ cells per well. Cultures were maintained in a humidified incubator at 37°C with a 95% air/5% CO_2_ atmosphere. At 2 days *in vitro* (DIV), hippocampal cultures were changed to neurobasal media (GIBCO) and the compound was added to the media. The cultures were fixed for immunocytochemistry at 5 DIV for neurite outgrowth analysis, day 9 DIV for staining with anti-synapsin 1 and 15 DIV for staining with anti-PSD95. Data on neurite outgrowth were analyzed by one-way ANOVA with Dunnet’s post hoc test and a t-test was used to analyse data on synaptic puncta. *P*<0.05 is considered as significant.

For biochemical analysis, neurons were treated with the anti-mitotic drug cytosine-β-D-arabinofuranoside (Ara C) to eliminate dividing astrocytes and used at 9–15 days after plating. The culture plates were washed with cool PBS and then added 200 µl lysis buffer over ice. Samples were vortexed and centrifuged at 13,000 rpm for 10 min, the supernatant was collected and stored in −80°C. Data on western blot were analyzed by t test. *P*<0.05 is considered as significant.

### Preparation of Organotypic Brain Slices

Hippocampal slices were prepared from postnatal day 5 (P5) mice and cultured according to the standard interface method described by De Simoni and Yu [48] with minor modifications. The hippocampi were dissected and placed in 4°C Grey’s Balanced Salt Solution supplemented with Glucose, then sliced into 400 µm -thick slices using a McIlwain tissue chopper. The slices with DG were identified and selected under Zeiss Stemi DV4 Stereomicroscope (Carl Zeiss Pte Ltd, Singapore) and transferred onto a porous membrane inserts (Millipore), placing 6 slices/insert and maintained in an incubator at 5% CO_2_/air at 37°C. The slice culture medium consisted of 50% Minimal Essential Medium with Glutamax (GIBCO), 25% Earle’s balanced salt solution, 25% heat-inactivated horse serum, Penicillin-Streptomycin 50 µg/ml, and glucose (6.5 mg/ml). The medium was changed every second day. On day 7 the culture medium was changed to serum free condition and taurine was added to the medium on day 9 and throughout every medium change. EdU (10 µM) was added to the culture medium on day 11 for 48 hrs and the slices were fixed on day 17 using 4% phosphate buffered paraformaldehyde and then processed for EdU, DCX and GFAP staining. Data were analyzed by one-way ANOVA with Dunnet’s post hoc test. *P*<0.05 is considered as significant.

### Animals

Time-mated C57BL/6 mice and Sprague-Dawley rats were purchased from Singhealth Experimental Medicine Centre (SEMC), Singapore, and housed in Specific Pathogen Free (SPF) animal facility at Duke-NUS Graduate Medical School, Singapore. All animals received water and food ad libitum. All animal procedures and applicable regulations of animal welfare were in accordance with IACUC guidelines. For labeling of proliferating cells in utero, a single injection of EdU (Invitrogen) dissolved in PBS were given at a dose of 50 mg/kg body weight on E13, 1 hr before the intra uterine taurine or vehicle injection.

### Immunocytochemistry, Immunohistochemistry, Confocal Imaging and Analysis

The following primary antibodies were used: goat anti-DCX (Santa Cruz, 1∶500), rabbit anti GFAP (Abcam, 1∶100). Rabbit anti-synapsin 1 (Abcam, 1∶500), mouse anti-PSD95 (Abcam, 1∶300). Images were acquired on a Zeiss LSM 7 ELYRA PS.1 system (Carl Zeiss, Pte. Ltd., Singapore) and analyzed using Zeiss Zen software. For analysis of the dendritic structure of neurons, the images were semi-automatically traced with NIH ImageJ using the NeuronJ plugin. The total dendritic length and branch number of each individual neuron were analyzed. A total of 200 neurons were analyzed per group. The synapse puncta was calculated as the number per µm. Data are presented as percentages normalized to the control (100%) ± SEM and at least 15 different neurons were quantified for each group and at least three repeated individual experiments were done.

For immunohistochemical analysis of brain slice cultures, slices were cryoprotected in 20% and the 30% sucrose solutions over 2 days. Next, the slices were embedded in an OTC compound and sectioned on a cryostat at thickness of 30 µm. After permeabilizing and blocking with serum, the sections were incubated with EdU staining reaction mix (Invitrogen) for 1 hr. Then the sections were incubated with antibodies against DCX and GFAP overnight at 4°C. The sections were then washed and incubated with secondary antibodies.

To determine the number of EdU positive cells in the granule cell layer (GCL) including the subgranular zone and the hilus of cultured slices, stacks of optical sections were taken under a confocal laser-scanning microscope using a 40X objective. For quantification, a square of 250 µm^2^ was randomly placed on three different locations of the DG. The cells in each square were counted and the three counts were averaged/section. Adjacent sections were not used for cell counting to avoid double counting. A total of 5 sections per cultured slice were used, and each experimental group consisted of 15–17 cultured slices from 3 independent experiments. Double-labeled cells for EdU and DCX or GFAP were visualized and counted with a 63 X oil immersion objective using Zeiss LSM 710 confocal system (Carl Zeiss Pte Ltd, Singapore). The number of EdU-labeled cells that expressed DCX or GFAP was determined by counting a minimum of 35 EdU-labeled cells on the sections spanning the entire dentate gyrus as described previously. The extent of colocalization was validated by viewing cells on three planes (X, Y, and Z) using Z-plane sectioning. Cells single labeled for EdU or double labeled for EdU/DCX or EdU/GFAP were counted. The percentage of EdU cells double labeled for DCX or GFAP was calculated by dividing the number of double-labeled cells by the total number of EdU cells and multiplied by 100. Data are presented as percentages normalized to the control (100%) ± SEM. Data were analyzed by t test. *P*<0.05 is considered as significant.

For staining of embryonic brain, at E17, brains were fixed in 4% paraformaldehyde. Cryostat sections (20 µm) of the brain were cut, mounted onto gelatinized slides and processed for EdU chemistry. EdU staining is performed as described for the cultured hippocampal slices. To determine the EdU intensity in the dentate gyrus, 6 sections (250 µm apart) were selected from identical areas of hippocampus of vehicle or taurine injected embryos (n = 6). Images were taken with Zeiss LSM 710 confocal system (Carl Zeiss Pte Ltd, Singapore) using a 40X objective. For quantification, a square of 250 µm^2^ was randomly placed on three different locations of the DG and images were taken maintaining the same imaging settings for all conditions. The fluorescence was quantified using image J. For each slice, three squares were analyzed and the three values were averaged/slice. Data are presented as percentages normalized to the control (100%) ± SEM and statistical analysis was done using t test. *P*<0.05 is considered as significant.

### Sodium Dodecylsulfate Polyacrylamide Gel Electrophoresis (SDS-PAGE) and Western Blot Analysis

Protein concentrations were determined using the BioRad Protein Assay. Dye Reagent Concentrate (Bio-Rad; Hercules, CA, USA). Equal amounts of samples were separated by SDS-PAGE. Following electrophoresis, the gel was transferred to a polyvinyldifluoride (PVDF) membrane (Millipore, Bedford, MA, USA), blocked with 5% skim milk in PBST (0.1 M PBS and 0.1% Tween 20), then incubated with the desired antibody at 4°C overnight (1∶5000 for the anti-p44/42 antibody (Cell Signaling), 1∶1000 for the anti-Phospho-P44/42 antibody (Cell Signaling), 1∶1000 for synapsin 1 (Abcam), 1∶300 for PSD 95 (Abcam), and 1∶5000 for the anti-α-tubulin antibody (Sigma). After three 10-min washes in PBST, the membranes were incubated with peroxidase-conjugated donkey anti-rabbit, goat anti-mouse IgG (Promega), at a 1∶5000 dilution for 1 h at room temperature (RT) and washed six times with PBST. The immunoreactive bands were visualized using an enhanced chemiluminescence (ECL) method. The data were analyzed using image-J software. One-way ANOVA with Dunnet’s post hoc test was used to analyse data on ERK1/2 and a t test was used to analyze western blot on synaptic proteins. *P*<0.05 is considered as significant.
